# Antioxidant Astaxanthin Co-Treatment Protects Zebrafish from Dimethomorph-Induced Cardiovascular Toxicity

**DOI:** 10.3390/ijms27073211

**Published:** 2026-04-01

**Authors:** Chia-Chen Wu, Ferry Saputra, Ross D. Vasquez, Marri Jmelou M. Roldan, Yu-Heng Lai, Chung-Der Hsiao, Chih-Hsin Hung

**Affiliations:** 1 Institute of Biotechnology and Chemical Engineering, I-Shou University, Kaohsiung 84001, Taiwan; maxwu02@gmail.com; 2 Division of Thoracic and Cardiovascular Surgery, Department of Surgery, Kaohsiung Chang Gung Memorial Hospital, Chang Gung University College of Medicine, Kaohsiung 833, Taiwan; 3 Department of Chemistry, Chung Yuan Christian University, Taoyuan 320314, Taiwan; ferrysaputratj@gmail.com; 4 Department of Pharmacy, Faculty of Pharmacy, University of Santo Tomas, Manila 1015, Philippines; rdvasquez@ust.edu.ph (R.D.V.); mmroldan@ust.edu.ph (M.J.M.R.); 5 Research Center for the Natural and Applied Sciences, University of Santo Tomas, Manila 1015, Philippines; 6 The Graduate School, University of Santo Tomas, Manila 1015, Philippines; 7 Department of Chemistry, Chinese Culture University, Taipei 11114, Taiwan; lyh21@ulive.pccu.edu.tw; 8 Department of Bioscience Technology, Chung Yuan Christian University, Taoyuan 320314, Taiwan; 9 Research Center for Aquatic Toxicology and Pharmacology, Chung Yuan Christian University, Taoyuan 320314, Taiwan

**Keywords:** dimethomorph, cardiovascular toxicity, zebrafish, antioxidant

## Abstract

Dimethomorph (DIM) is a commonly applied morpholine fungicide, yet its potential toxicity to non-target organisms raises significant environmental concerns. This study aims to elucidate the toxicological effects of DIM on zebrafish, with a particular focus on its cardiovascular impacts. Zebrafish embryos were exposed to a range of DIM concentrations for 48 h, and their cardiac and vascular performance was meticulously assessed to determine the extent of cardiovascular toxicity. The findings revealed notable cardiac hypertrophy, evidenced by a substantial enlargement in heart size, alongside a dose-dependent reduction in heart rate. These observations suggest direct impairment of cardiac function following DIM exposure. To further investigate the molecular underpinnings of these effects, gene expression analysis was conducted using quantitative real-time PCR (qRT-PCR). The results demonstrated significant alterations in the expression of key genes associated with cardiovascular development and function, providing mechanistic insights into DIM’s toxic effects. In addition to cardiac abnormalities, DIM exposure led to a significant increase in the metabolic rate of the zebrafish embryos, indicating a potential disruption in energy homeostasis. To explore possible protective measures, a rescue experiment was performed using Astaxanthin, a potent antioxidant. Notably, Astaxanthin treatment partially mitigated the observed cardiac and metabolic phenotypes, suggesting its potential as a therapeutic agent to counteract DIM-induced toxicity. In summary, this study provides compelling evidence of the cardiovascular toxicity of DIM in zebrafish, highlighting its potential to disrupt cardiac function and metabolic regulation. The observed effects underscore the importance of re-evaluating the environmental impact of DIM and emphasize the need for further research to fully understand its mechanisms of toxicity. The findings also suggest that Astaxanthin (AST) could serve as a protective agent against DIM-induced toxicity, opening avenues for future studies aimed at mitigating the adverse effects of this widely used fungicide. This research study contributes to the growing body of knowledge on the environmental and health risks associated with pesticide use, advocating for more stringent regulations and safer alternatives.

## 1. Introduction

Dimethomorph (DIM) is a synthetic fungicide in the morpholine chemical group. It inhibits fungal growth by disrupting the formation of the fungal cell wall. It is widely used in agriculture to protect crops like grapes, potatoes, tomatoes, and other vegetables from fungal diseases [1]. In Taiwan, DIM is primarily applied in grape production to prevent fungal infections [2]. The half-life of DIM in soil can reach up to 33 days, with residues detected in fruits such as cucumbers and grapes [3]. Studies indicate that DIM can accumulate in non-target organisms, such as ground beetles [4,5]. Furthermore, evidence suggests that DIM leaches into nearby water bodies, raising concerns about its environmental impact on aquatic organisms [6]. The persistence of DIM in soil and water has been linked to adverse effects on non-target species. For example, DIM has been shown to significantly inhibit the growth of duckweed [7]. Teather et al. reported that the commercial pesticide Acrobat TM^®^ (containing 9% DIM) causes abnormal development and behavioral changes in Japanese medaka [8]. In soil, DIM exposure at 100 mg/kg was found to alter enzymatic functions related to reactive oxygen species (ROS) production in earthworms [9]. Similarly, DIM caused some developmental defects and impaired locomotion in zebrafish larvae at concentrations as low as 2.96 ppm, while higher concentrations (>9.28 ppm) significantly reduced heart rate [10]. Additionally, perinatal exposure to DIM at 180 mg/kg in female rats disrupted plasma hormone profiles and ovarian gene expression [11]. These findings highlight the toxic effects of DIM on non-target organisms warranting further investigation into its mechanisms of action. The widespread use and environmental persistence of DIM raise significant concerns about its potential toxicity to non-target species. Studies have demonstrated its adverse effects on various organisms, including earthworms, duckweed, Japanese medaka, and rats, highlighting its potential to disrupt biological systems across ecosystems. Despite this, the detailed mechanisms underlying DIM’s toxicological effects remain poorly understood, particularly regarding its impact on aquatic organisms. Given its propensity to leach into water bodies, it is essential to assess DIM’s toxicity in a controlled aquatic environment. The toxic effects of DIM on non-target organisms highlight the need for further investigation into its specific mechanisms of action, particularly its impact on cardiovascular functions, genetic alterations, and behavioral outcomes. Studies should focus on how DIM influences cardiac performance and vascular integrity at the molecular level and on its role in altering the expression of key cardiovascular-related genes.

Zebrafish (*Danio rerio*), an established vertebrate model for toxicological research, provide a powerful system for elucidating the effects of waterborne toxicants like DIM on cardiovascular functions. Zebrafish exhibit genetic homology with humans, rapid development, and sensitivity to environmental changes [12]. They offer a high-throughput, cost-effective system for assessing the impact of toxicants on biological processes, including cardiovascular and neurobehavioral functions [13]. The transparency of zebrafish embryos allows for direct observation of organ function, such as heartbeats and blood flow, using advanced imaging techniques [12,14]. Furthermore, zebrafish exhibit cognitive and socio-affective behaviors analogous to humans, making them suitable for neurobehavioral studies [15]. Their sensitivity to waterborne toxicants also makes them an ideal model for assessing the environmental impacts of pesticides and fungicides [16].

This study aims to investigate the toxicity of DIM by using zebrafish as a model organism. Preliminary findings indicate that acute DIM exposure induces hyperactivity in zebrafish, prompting further exploration of potential cardiovascular toxicity. To achieve this, zebrafish were exposed to varying concentrations of DIM, and their cardiovascular function was evaluated through cardiac and vascular performance parameters. Gene expression analysis was conducted to assess changes in cardiovascular-related markers. To explore potential mechanisms of toxicity, a rescue experiment using the antioxidant AST was also conducted. Given the limited research on the detailed toxicological profile of DIM, this study provides valuable insights into its adverse effects on non-target animal models.

## 2. Results

### 2.1. Acute Toxicity Test

**Figure 1:**
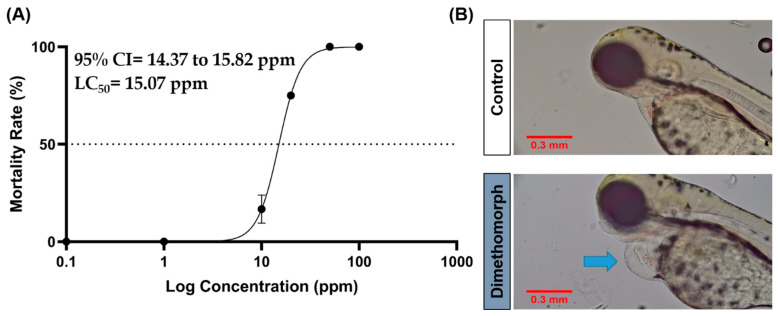
Acute toxicity test (LC_50_) results of zebrafish larvae exposed to DIM for 96 h (**A**) and comparison of cardiac chamber size of control and zebrafish treated with 10 ppm of Dimethomorph (horizontal dotted line = LC_50_ point). (**B**) Blue arrow indicates the presence of cardiac edema in DIM-treated group. Scale bar = 0.3 mm.

An acute toxicity test was conducted to determine the relative toxic concentration of DIM for further testing. The results show that the 96 h lethal concentration 50 (LC_50_) of DIM was 15.07 ppm (Figure 1A). This finding is consistent with previous research which reported an LC_50_ of 11.84 mg/L for 96 h exposure, with exposure beginning 4 h post-fertilization (hpf) [10]. Additionally, this study observed an increase in cases of cardiac edema (>80%) at 96 h post-exposure, starting at a concentration of 10 ppm. All treated fish died within 24 h of exposure at concentrations starting from 50 ppm (Figure 1B). These findings indicate that DIM falls into the category of “slightly toxic” according to the US Environmental Protection Agency [EPA] [17]. The concentrations used in further assays were selected based on the sub-lethal concentration obtained from the LC_50_ calculations. Based on this, concentrations of 0.5, 5, and 10 ppm were selected for further testing.

### 2.2. Cardiovascular Performance Assay

**Figure 2:**
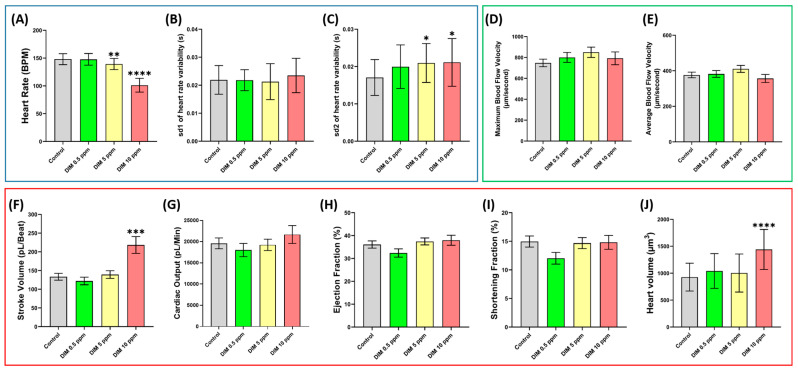
Cardiovascular performance assay results for DIM (Dimethomorph)-exposed zebrafish. (**A**) Heart rate, (**B**) sd1 of heart rate variability, (**C**) sd2 of heart rate variability, (**D**) stroke volume, (**E**) cardiac output, (**F**) ejection fraction, (**G**) shortening fraction, (**H**) maximum blood flow velocity, (**I**) average blood flow velocity, and (**J**) heart volume. Data distribution was analyzed using Anderson–Darling test, D’Agostino–Pearson test, Shapiro–Wilk test, and Kolmogorov–Smirnov test. As the data passed the normality tests (α = 0.05), one-way analysis of variance (ANOVA) followed by Dunnett’s multiple comparison test was used to compare the means among groups. Data are presented as means ± standard deviations (SDs). Blue box shows parameter related to cardiac rhythm, while red and green boxes show parameters related to cardiac physiology and vascular performance respectively. * *p* < 0.05, ** *p* < 0.01, *** *p* < 0.001, and **** *p* < 0.0001. Each control and treatment group originally consisted of 16 fish, and the experiment was conducted in duplicate. Due to mortality, total sample size (*n*) varied among treatment groups: *n* Control= 30, *n* DIM 0.5 ppm= 32, *n* DIM 5 ppm= 32, and *n* DIM 10 ppm= 31.

This study observed notable alterations in zebrafish cardiovascular function, primarily in the cardiac rhythm, stroke volume, and heart volume. A significant dose-dependent decrease in heart rate was detected, beginning at 5 ppm (*p* = 0.0044) (Figure 2A). This was accompanied by an increase in overall heartbeat irregularity at 5 ppm (*p* = 0.0199) and 10 ppm (*p* = 0.0154) (Figure 2C). Significant changes in stroke volume were also evident at 10 ppm (*p* < 0.0001) (Figure 2F). At the 10 ppm concentration, the ventricular chamber was significantly enlarged compared with the control group (*p* < 0.0001), suggesting cardiac toxicity induced by DIM exposure (Figure 2J); meanwhile no significant changes were observed in cardiac output, ejection fraction, and shortening fraction (Figure 2G–I). Similarly, no observable changes were detected in the maximum or average blood flow velocity in the dorsal aorta following DIM treatment (Figure 2D,E).

### 2.3. Metabolic Rate Assessment

**Figure 3:**
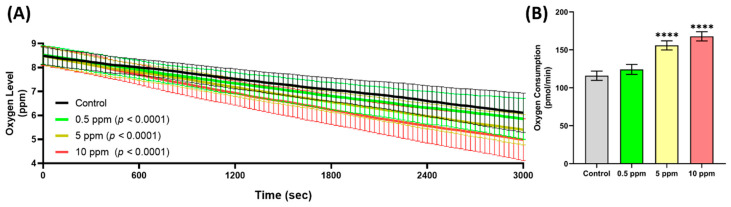
Metabolic rate assessment results for DIM (Dimethomorph)-exposed zebrafish. (**A**) Overview of oxygen level inside the well during the entire test period and (**B**) total oxygen consumption of zebrafish in whole test duration (50 min). Data distribution for both endpoints was analyzed using Anderson–Darling test, D’Agostino–Pearson test, Shapiro–Wilk test, and Kolmogorov–Smirnov test. As the data passed the normality tests (α = 0.05), statistical variance for chronological oxygen consumption was calculated using two-way analysis of variance (ANOVA) with Geisser–Greenhouse correction followed by Dunnett’s multiple comparison post hoc test to compare each treatment group to the control group; data are presented as means ± SDs. Meanwhile, total oxygen data were analyzed using one-way ANOVA with Dunnett’s multiple comparison post hoc test to compare each treatment group to the control group and are presented as means ± SDs. **** *p* < 0.0001. Each control and treatment group originally consisted of 23 fish, and the experiment was conducted in triplicate. Due to mortality, *n* varied among treatment groups: *n* Control = 63, *n* 0.5 ppm = 65, *n* 5 ppm = 63, and *n* 10 ppm = 64.

Following the cardiovascular assessment, a follow-up study was conducted to evaluate the metabolic rate by analyzing oxygen consumption. The results revealed significant alterations in oxygen consumption throughout the 50 min test period, with a marked reduction in free oxygen levels in the chamber being observed at all tested concentrations (*p* < 0.0001). This reduction was evident as early as 10 min into the test (Figure 3A). Additionally, the data showed a significant dose-dependent increase in total oxygen consumption by the end of the test (Figure 3B).

### 2.4. Gene Expression Assessment

**Figure 4:**
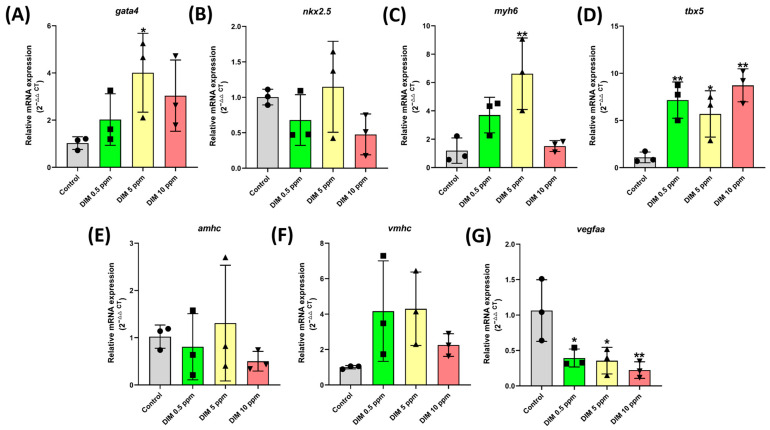
Expression of marker gene related to cardiovascular development in DIM (Dimethomorph)-exposed zebrafish. (**A**) *gata4*, (**B**) *nkx2.5*, (**C**) *myh6*, (**D**) *tbx5*, (**E**) *amhc*, (**F**) *vmhc*, and (**G**) *vegfaa*. Data distribution for both endpoints was analyzed using Anderson–Darling test, D’Agostino–Pearson test, Shapiro–Wilk test, and Kolmogorov–Smirnov test. As the data passed the normality tests (α = 0.05), one-way analysis of variance (ANOVA) with Dunnett’s multiple comparison post hoc test was used to compare each treatment group to the control group and are presented as means ± SDs. * *p* < 0.05 and ** *p* < 0.01. Each control and treatment group originally consisted of 1 sample and the experiment was conducted in triplicate. *n* = 3.

To evaluate the impact of DIM on cardiovascular development, gene expression analysis was performed, focusing on genes related to cardiovascular function. The results showed significant alterations in the expression of T-box transcription factor 5 (*tbx5*) at all tested concentrations, with *p*-values of 0.0087, 0.0356, and 0.0022 for 0.5, 5, and 10 ppm, respectively (Figure 4A). At 5 ppm, significant changes were observed in GATA binding protein 4 (*gata4*) (*p* = 0.0482) and myosin heavy-chain cardiac muscle alpha (*myh6*) (*p* = 0.0055) expression (Figure 4A,C,D). Furthermore, vascular endothelial growth factor Aa (*vegfaa*) expression was significantly altered across all concentrations tested, with *p*-values of 0.0291, 0.0233, and 0.0090 for 0.5, 5, and 10 ppm, respectively, suggesting potential vascular development issues (Figure 4G). On the other hand, NK2 homeobox 5 (*nkx2.5*), atrial myosin heavy chain (*amhc*), and ventricular myosin heavy chain (*vmhc*) (Figure 4B,E,F) did not show any alteration post-DIM treatment. Collectively, these findings provide strong evidence supporting the cardiovascular toxicity of DIM in zebrafish larvae.

### 2.5. Rescue Experiment Using Astaxanthin

**Figure 5:**
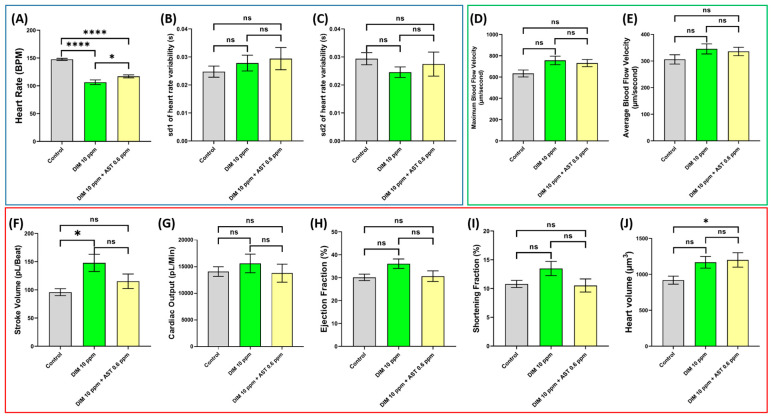
Cardiovascular performance assay results of groups treated with DIM (Dimethomorph) at 10 ppm and DIM 10 ppm + AST 0.6 ppm (Astaxanthin). (**A**) Heart rate, (**B**) sd1 of heart rate variability, (**C**) sd2 of heart rate variability, (**D**) maximum blood flow velocity, (**E**) average blood flow velocity, (**F**) stroke volume, (**G**) cardiac output, (**H**) ejection fraction, (**I**) shortening fraction, and (**J**) heart volume. Data distribution for both endpoints was analyzed using Anderson–Darling test, D’Agostino–Pearson test, Shapiro–Wilk test, and Kolmogorov–Smirnov test. As the data passed the normality tests (α = 0.05), one-way analysis of variance was conducted to compare the means among groups, followed by Tukey’s multiple comparison test for pairwise comparisons. All data are presented as means ± SDs. Blue box shows parameters related to cardiac rhythm, green box shows parameters related to vascular performance, and red box shows parameters related to cardiac physiology. ns = not significant, * *p* < 0.05 and **** *p* < 0.0001. Each control and treatment group originally consisted of 16 fish, and the experiment was conducted in duplicate. Due to mortality, *n* varied among treatment groups: *n* Control = 31, *n* DIM = 30, and *n* DIM + AST = 29.

Astaxanthin (AST), a potent antioxidant, has previously been shown to mitigate the effects of pesticides by neutralizing reactive oxygen species (ROS). In this study, partial alleviation of DIM-induced damage was observed in several areas. The heart rate of zebrafish exposed to both DIM and AST was significantly higher than that of those treated with DIM (*p* = 0.0428). However, the heart rate in the DIM + AST group remained significantly lower than that of the control group (*p* < 0.0001) (Figure 5A), unlike sd1 and sd2, which signifies that heart rate variability was not significantly altered between treatment groups and control (Figure 5B,C). In the vascular performance endpoints, no significant effects were observed after exposure to DIM and DIM + AST in comparison to the control group (Figure 5D,E). A rescue effect was observed in stroke volume: while the DIM-only group showed a significant increase in stroke volume, co-exposure with AST reduced this increase to a statistically non-significant difference compared with the control group (*p* = 0.116) (Figure 5F). However, exposure to DIM + AST seemed to increase heart size significantly (*p* = 0.0.392) in comparison with the control group but not statistically significantly compared to the DIM-only group (*p* = 0.9569) (Figure 5J). Meanwhile, the other observed endpoints in the cardiac physiology endpoint group did not show any significant changes post-exposure to DIM and DIM + AST (Figure 5G–I).

## 3. Discussion

**Figure 6:**
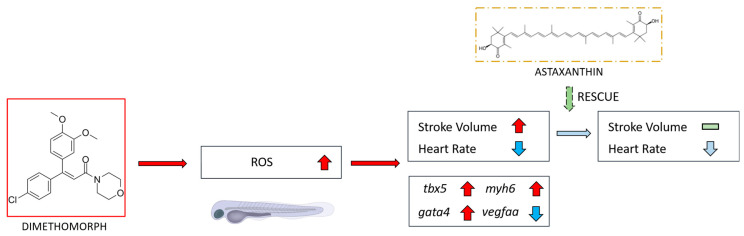
Overview of the potential toxicity of DIM (Dimethomorph) and its hypothesized mechanism of action. This study suggests that DIM exposure induces ROS production, which leads to alterations in the genes involved in cardiovascular development and function. Furthermore, Astaxanthin (AST) was found to partially alleviate the damage caused by DIM exposure. Red upward arrow represents increased activity/expression, blue downwardarrow represents decreased activity/expression, while grey downward arrow represents decreased activity at lower degree.

Our study provides clear evidence that DIM induces toxicity in zebrafish larvae. The results demonstrate that DIM exposure leads to cardiac edema and cardiomegaly, as well as a significant dose-dependent increase in stroke volume and a decrease in heart rate. Additionally, we observed a significant increase in oxygen consumption, which is commonly associated with elevated production of reactive oxygen species (ROS) as a byproduct (Figure 6) [18]. Supporting this, the qRT-PCR analysis revealed significant alterations in the expression of genes related to cardiovascular development. While AST partially alleviated DIM-induced cardiac toxicity, it did not fully restore normal cardiac function. Based on these findings, we conclude that DIM toxicity likely involves multiple pathways beyond ROS production.

In this study, cardiomegaly and cardiac edema were observed, accompanied by a significant decrease in heart rate in zebrafish exposed to DIM. To our knowledge, this is the first study to report cardiomegaly in a morpholine-related study using zebrafish. Cardiomegaly is an enlargement of the heart, which can result from either hyperplasia or hypertrophy [19,20]. This condition is often associated with several heart-related problems, including high blood pressure, myocardial infarction, arrhythmia, and other underlying causes [21]. In a previous study, cardiomegaly was observed in rabbits following daily consumption of fenproprimorph at a dose of 60 mg/kg body weight, which resulted in dilation of the right heart chamber [22]. Furthermore, cardiac edema has been reported in zebrafish exposed to DIM. Fan et al. documented cardiac edema in zebrafish after DIM exposure, accompanied by a significant decrease in the heart rate, beginning at a concentration of 9.28 ppm [10]. These findings collectively suggest that DIM exposure may lead to cardiotoxicity in zebrafish.

Our observation suggests a significant increase in metabolic rate, as evidenced by the elevated oxygen consumption. This increase may be attributed to myocardial oxygen demand. Myocardial oxygen consumption is influenced by preload, afterload, contractility, and heart rate. Given the enlargement of the heart in DIM-exposed fish, the demand for myocardial contraction likely increases [23]. These factors likely explain the significant increase in the oxygen consumption observed in the DIM-exposed group.

This study observed significant alterations in the expression of cardiovascular-related genes, particularly in *gata4*, *myh6*, *tbx5*, and *vegfaa*. Overexpression of *gata4*, *myh6*, *and tbx5* suggests potential issues in cardiac development. GATA4 is a transcription factor crucial to the formation of cardiac structures, including morphogenesis and cardiac cell differentiation, through BMP and Wnt signaling pathways [24,25]. In humans, mutations in *gata4* can lead to several cardiac disorders, including dilated and hypertrophic cardiomyopathy, atrial and ventricular septal defects, and bicuspid aortic valve [24]. Similarly, MYH6 plays a vital role in cardiac development. Although primarily expressed in the atrium, MYH6 is also found in the ventricle during early development, which is responsible for the formation of myosin heavy chains that are important for heart contractility [26]. Mutations in *myh6* have been linked to hypertrophic and dilated cardiomyopathy [27]. TBX5, a member of the T-box transcription factor family, is essential to cardiac and forelimb development. It regulates early heart development by controlling ventricular wall thickness and the formation of the atrial conduction system [28,29]. As a key regulator, TBX5 interacts with multiple signaling pathways, including Wnt, BMP, Notch, TGF-β, and Nodal signaling [28,30,31,32]. On the other hand, VEGFAA encodes VEGFA, which is crucial to angiogenesis. VEGFA binds to VEGFR-1 and VEGFR-2 on endothelial cells, promoting cell proliferation and migration to facilitate the formation of new blood vessels [33,34]. Downregulation of *vegfaa* expression could compromise blood vessel structure, leading to various cardiovascular-related issues [4,35].

A rescue experiment was conducted using the strong antioxidant Astaxanthin (AST) due to increased metabolic rate and oxygen consumption, which may lead to elevated ROS production. Astaxanthin is a red pigment belonging to the carotenoid group, commonly found in algae, salmon, and crustaceans, which is responsible for their red color. Approved as a food additive by the United States Food and Drug Administration in 1987, AST is now widely used as a dietary supplement [36].

In our study, AST partially alleviated the damage caused by DIM, which aligns with previous research where AST reduced the toxic effects of various chemicals and pesticides in zebrafish. Recent studies showed that exposure to DIM resulted in heightened ROS levels in transgenic zebrafish larvae. Heightened ROS levels were especially apparent in the heart area of the zebrafish larvae in a dose-dependent manner, and the increase in ROS levels was most likely related to vascular and cardiac developmental defects observed in the study [37], which were also observed to some degree in this study (cardiomegaly and cardiac edema). AST as a potent antioxidant played a role in alleviating the damage caused by heightened ROS levels due to DIM exposure by scavenging and inhibiting ROS, as well as free radicals. The capability of AST to alleviate ROS damage has been previously demonstrated: Wei et al. (2021) showed that AST could partially mitigate the cardiotoxicity induced by iprodione due to increased ROS levels in zebrafish larvae by regulating ROS levels [38]. Similarly, Huang et al. (2020) reported that AST alleviated cardiac damage caused by the combination of oxadiazone and butachlor exposure in zebrafish larvae, which also increased ROS levels in zebrafish [39]. Chen et al. (2023) also demonstrated that AST reduced ROS accumulation induced by methyl parathion, which in turn mitigated cardiac edema in zebrafish larvae [40]. These studies suggest that ROS accumulation plays a significant role in DIM-induced toxicity, as AST partially alleviates the observed damage. Thus, these studies support our findings on alleviation of DIM effects. On the other hand, it is important to note that using 0.6 ppm AST and 10 ppm DIM for a 48 h exposure period in our experiment may not fully replicate the actual concentrations of DIM found in ecological waters, as recent research showed the estimated highest concentration found in water to reach up to 0.639 mg a.i./L 41 days after application [6]. Therefore, the results, while insightful, may not wholly reflect the environmental relevance of DIM toxicity in natural habitats. Nevertheless, these findings are important as evidence and a basis for future studies to understand the mechanisms of DIM-induced toxicity and explore potential interventions to mitigate its effects.

Our rescue experiment demonstrated the capability of AST to protect cardiac function from DIM at high concentrations (10 ppm); this partial alleviation might indicate that DIM toxicity is not limited to ROS production. While many studies have reported a significant increase in ROS production after pesticide exposure, ROS may not always be the primary cause of damage but rather a secondary effect triggered by stress signals [38,39,41]. A recent study suggested an approach focusing on thyroid hormone, which regulates heart development, angiogenesis, and overall embryonic growth [37]. Regulating this hormone might yield better results compared with astaxanthin, which only regulates ROS levels, and combining thyroid treatment and astaxanthin might also be a good approach for future study. This gap in our study presents an interesting avenue for future research, which could help deepen our understanding of the mechanisms underlying DIM toxicity.

## 4. Materials and Methods

### 4.1. Animal Care and Maintenance

In this study, AB wild-type zebrafish strains were acquired from Academia Sinica (TZCAS) and sustained in a filtered water system. The water temperature was maintained at 26 °C under a 10 h dark and 14 h light cycle each day. The fish were alternately fed with fresh brine shrimp and frozen *Daphnia*. Two males and one female adult zebrafish were placed into a breeding chamber overnight to obtain eggs. The following morning, the separator was removed, and the eggs were collected one hour later. To synchronize fertilization time, the time of egg collection was designated as 0 h post-fertilization. Before exposure, the eggs were kept at 28 °C until 24 h post-fertilization. During exposure, the eggs were continuously kept under the same conditions until the day of the experiment. All protocols and procedures were approved by the Committee of Animal Experimentation of Chung Yuan Christian University (approval number CYCU110016, approved on 1 December 2021).

### 4.2. Chemical Preparation and Zebrafish Exposure

DIM with a purity of 97% was purchased from BiDe Pharmaceutical Technology Co., Ltd. (Shanghai, China). The stock solution was diluted using dimethyl sulfoxide (DMSO) (TEDIA, Fairfield, OH, USA) to a concentration of 5000 ppm. Astaxanthin (AST) was purchased from Sigma-Aldrich (St. Louis, MO, USA), and the stock solution was diluted using DMSO to a concentration of 1000 ppm. Both stock solutions were further diluted to the desired working concentrations using ddH_2_O. For all experiments, DMSO at a concentration of 0.2% was used as the solvent control. Zebrafish were exposed to DIM at 0.5, 5, and 10 ppm. Treatment on zebrafish larvae started at 24 h post-fertilization (hpf) for 48 h before observation in various assays. DIM concentration and treatment exposure are the same for all assays unless stated otherwise.

### 4.3. Malformation and Acute Toxicity Testing

For acute toxicity testing, eight zebrafish eggs, appearing in good condition under a Nikon stereo miscroscope (SMZ-1, Nikon, Japan, Tokyo) at 24 hpf, were selected and placed into 96-well plates. DIM was tested at concentrations of 0.1, 1, 10, 20, 50, and 100 ppm to determine the LC50 of the compound, following the modified OECD Guideline No. 236. Observations were made every 24 h, and the LC_50_ 96 h after exposure was used for further testing. The experiment was done in three biological replications.

### 4.4. Cardiac Toxicity Assay

The cardiac toxicity assay was performed by calculating stroke volume (SV), cardiac output (CO), ejection fraction (EF), shortening fraction (SF), heart rate (HR), and heart rate variability, which includes the standard deviation 1 (sd1) and 2 (sd2), following the protocol developed by Hoage et al. [42] and our modified protocol [43,44]. In brief, zebrafish larvae at 72 hpf were placed on a glass slide with 3% methylcellulose as the mounting solution. The fish were positioned to show a lateral view, with the head facing left and the belly facing down. The heart chamber was recorded with a high-resolution 4K Charge-coupled device (CCD) camera (XP4K8MA, ToupTek, Hangzhou, China) mounted on an upright microscope (EX20, SOPTOP, Taipei, Taiwan). The recording was conducted under 10× magnification for one minute, focusing on the ventricle chamber. Cardiac performance was assessed by calculating the short and long axis of the ventricle during the end systolic (ESV) and diastolic phase (EDV), assuming the heart had a spheroid shape. The calculations were done using the following formulas:

(1) SV=EDV−ESV

(2) CO=SV×HR

(3) $$\mathrm{EF}\left(\%\right)=\frac{\mathrm{SV}}{\mathrm{EDV}}\times 100\%$$

(4) $$\mathrm{SF}\left(\%\right)=\frac{\mathrm{Ds}\left(\mathrm{EDV}\right)-\mathrm{Ds}\left(\mathrm{ESV}\right)}{\mathrm{Ds}\left(\mathrm{EDV}\right)}\times 100\%$$

Heart rate calculation was done using the Time Series Analyzer V3 plugin on ImageJ Software v1.52 under FIJI distribution (https://imagej.nih.gov/ij/plugins/time-series.html, accessed on 23 May 2024). The software tool detects changes in pixel brightness intensity over the selected region of interest throughout the entire video. Based on this data, the timing of the ventricle beat is determined and used to calculate the heart rate. Heart rate variability was calculated using the ventricle beat interval with the Poincare plot plugin in OriginPro 2019 Software v9.6.5.169 (Originlab Corporation, Northampton, MA, USA). Approximately 16 fish were used for each group, and the experiment was done in duplicate [45].

### 4.5. Vascular Toxicity Assay

The vascular toxicity assay was performed by calculating the maximum and the average blood flow velocity in the dorsal aorta, following the protocol developed by Santoso et al. (2019) [46]. Zebrafish at 72 hpf were placed into two-centimeter Petri dishes with the same position as the previous cardiac performance assay. The fish were mounted using 3% methylcellulose, and the recording was done using a high-speed CCD camera (AZ Instrument, Taichung City, Taiwan) mounted on an inverted microscope (ICX41, Sunny Optical Technology, YuYao, China). To record the dorsal aorta area, a Hoffmann Modulating Lens with 40× magnification (Olympus, Tokyo, Japan) was used to achieve high clarity of the flowing blood cells, and recording was made at 200 frames per second. The TrackMate plugin in ImageJ was used to analyze the speed of blood cells in the dorsal aorta following exposure to DIM [47]. A total of 16 fish larvae were used for each treatment group, and the experiment was conducted in duplicate [45].

### 4.6. Metabolic Rate Analysis

The metabolic rate was assessed by calculating the oxygen consumption of the larvae after DIM exposure. This was done by placing zebrafish larvae into a specialized 24-well plate with an oxygen reader sensor on the top of the Sensor Dish Reader (SDR) (Loligo Systems, Viborg, Denmark). The oxygen concentration inside the Petri dishes was monitored periodically using MicroResp^®^ version 1.0.4 (Loligo Systems, Viborg, Denmark) software. Approximately 23 zebrafish larvae at 96 hpf were placed into the wells, along with 80 µL of DIM at the desired concentration. The oxygen concentration was monitored for 50 min, and the study was conducted in triplicate.

### 4.7. Gene Expression Analysis

Gene expression analysis was performed by conducting qRT-PCR on several genes. Approximately 50 live embryos were collected after exposure to DIM, and the total RNA was extracted using RNAzol reagent (Molecular Research Center, Inc. Cincinnati, OH, USA). The extraction began by pooling the fish into a 1.5 mL tube, and 1 mL per 50 mg tissue was added. The tissue was then homogenized using a bead blender for 15 min. About 0.4 mL of water was added per 1 mL of RNAZol used for homogenization, followed by vigorous shaking for 15 s and storage for 15 min at room temperature. The sample was then centrifuged at 12,000× *g* for 15 min, and the supernatant was transferred to a new tube. The sample was precipitated by adding 1 mL of isopropanol per 1 mL of supernatant, and the sample was stored for 10 min at room temperature. The sample was then centrifuged at 12,000× *g* for 10 min and washed by adding 75% ethanol. The washing procedure was performed twice with centrifugation at 8000× *g* for 3 min between each wash. The excess ethanol was removed after each wash, and the RNA was dissolved in ddH_2_O to obtain the concentration of 1–2 µg/mL. Gene expression was normalized to the relative expression of β-actin as an internal control, and the 2^−∆∆CT^ method was used to compare the expression of each gene with the internal control. The expression of seven genes related to zebrafish heart development was observed: ventricular myosin heavy chain (*vmhc*), atrial myosin heavy chain (*amhc*), vascular endothelial growth factor Aa (*vegfaa*), T-box transcription factor 5 (*tbx5*), NK2 homeobox 5 (*nkx2.5*), GATA binding protein 4 (*gata4*), and myosin heavy-chain cardiac muscle alpha (*myh6*). The sequences of the primers used for qRT-PCR are shown in Table 1. The experiment was done in triplicate, with 1 sample per replicate [45].

### 4.8. Rescue Experiment Using Antioxidant

The rescue experiment was conducted by co-exposing zebrafish to both DIM and AST. Approximately 16 zebrafish eggs were exposed to 0.6 ppm of AST at 24 hpf, and DIM at a concentration of 10 ppm was added 10 min after AST exposure. After 48 h of exposure, cardiac and vascular performance were assessed following the previously mentioned protocols. AST concentration was decided through a preliminary test at different concentrations: 0, 0.1, 0.2, 0.4, 0.6, 0.8, and 1 ppm. The preliminary test showed a reduction in activity at ≥0.8 ppm; thus 0.6 ppm was chosen for this study. The experiment was done in duplicate [45].

### 4.9. Statistical Analysis

All statistical analyses in this study were performed using GraphPad Prism 8.0.2 software (GraphPad Software Inc., La Jolla, CA, USA). Data distribution was assessed through normality tests using the Anderson–Darling test, the D’Agostino–Pearson test, the Shapiro–Wilk test, and the Kolmogorov–Smirnov test. The appropriate parametric or non-parametric statistical ANOVA test was selected based on the normality of the data distribution for each treatment group.

## 5. Conclusions

In conclusion, our study provides important insights into the potential toxicity of DIM exposure in zebrafish, an off-target organism. Notably, we observed significant physiological changes in cardiovascular performance, including a substantial decrease in heart rate, which were the most prominent effects. Moreover, rescue experiments further support the hypothesis that ROS accumulation plays a significant role in DIM-induced damage, as AST administration partially alleviates these effects. Our findings suggest that DIM toxicity is not limited to ROS production and regulation, and recent studies highlighted thyroid hormone to be an important target in DIM toxicity. Given the limited research addressing the toxicity of DIM in non-target organisms, this study contributes valuable knowledge to understanding DIM’s potential risks, particularly regarding its overuse and the possibility of DIM poisoning. The results also open avenues for future research into the mechanistic pathways involved, which could aid in developing effective treatments for DIM toxicity.

## Figures and Tables

**Table 1:** List of primers used for gene expression analysis.

Gene	Forward Primer (5′–3′)	Reverse Primer (5′–3′)
*vmhc*	F: GAAGAGGCAGAGGCATCACT	R: AATTGCGTTTGCTCTGCTCC
*amhc*	F: AAGCCACTACCGCCTCTCTA	R: TTTGAGGCAAGGTCGTCCAA
*vegfaa*	F: AAAAGAGTGCGTGCAAGACC	R: GACGTTTCGTGTCTCTGTCG
*tbx5*	F: ATTCGCCGATAACAAATGG	R: CGCCTTGACGATGTGGAT
*nkx2.5*	F: GTCCAGGCAACTCGAACTACTC	R: AACATCCCAGCCAAACCATA
*gata4*	F: TCCAGGCGGGTGGGTTTATC	R: TGTCTGGTTCAGTCTTGATGGGTC
*myh6*	F: CACCAGCAGACACTGGATG	R: GCTCCAAGTCCATTCTGAC

## Data Availability

The original contributions presented in this study are included in the article. Further inquiries can be directed to the corresponding authors.
